# Supplementary material for: Quantifying Phenology in the Deciduous Tree and Phytophagous Insect System: A Methodological Comparison
Source: Ecol Evol. 2025 Sep 2;15(9):e71821. doi: 10.1002/ece3.71821 (PMC12403115; doi:10.1002/ece3.71821)
Supplement: Supplementary file 1 — Appendix S1. [file ECE3-15-e71821-s001.pdf]

Quantifying phenology in the deciduous tree and phytophagous insect system: a methodological comparison – Supplementary Information

Authors: Lucy M. Morley[1,*], Sam J. Crofts[1], Ella F. Cole[1], Ben C. Sheldon[1]

[1]Edward Grey Institute of Field Ornithology, Department of Biology, University of Oxford, United Kingdom

Corresponding author:
* L. M. M. lucy.morley@biology.ox.ac.uk

Contents

Supplementary Table 1 (collection dates for field methods) 2

Supplementary Figure 1 (bud development scoring keys) 3

Supplementary Figure 2 (caterpillar identification keys) 4

Supplementary Table 2 (summary statistics of phenological metrics) 5

Supplementary Figure 3 (repeatability) 6

Supplementary Figure 4 (beech correlation matrix) 7

Supplementary Figure 5 (cross-trophic level model plots) 8

Supplementary Figure 6 (herbivory model plots) 9

Field Method	Start Date	End Date	Number of Sampling Days
Multispectral drone imaging	21st March	9th June	21-23
Bud scoring observations	25th March	25th May	20-21
Hemispherical canopy photography	25th March	8th June	16-22
Water traps	3rd May	20th June	14
Frass traps	9th May	15th June	11

Supplementary Table 1. Collection dates for each of the five field methods measuring the tree and caterpillar phenology of 170 trees in Wytham Woods, Oxfordshire, during spring 2023. Variation in the number of sampling days depends on flight/ground sampling area.

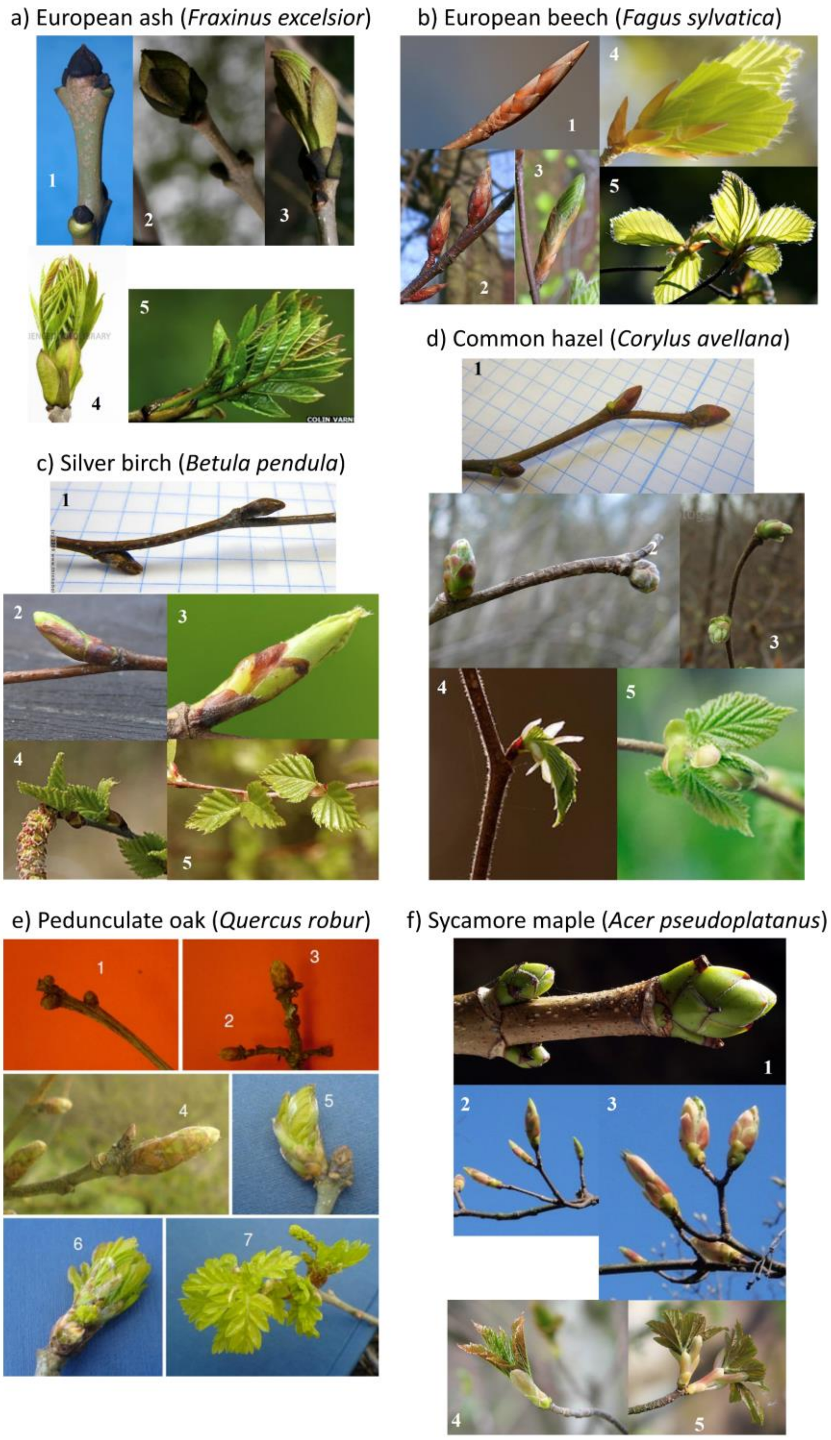

Supplementary Figure 1. Photographic reference keys used for scoring bud development, from 1 (no or minimal bud swelling), to 5 (for non-oaks; **a-d & f**) or 7 (for oaks; **e**) denoting full leaf. These are the same keys used in Cole & Sheldon (2017). Images taken from Google Images.

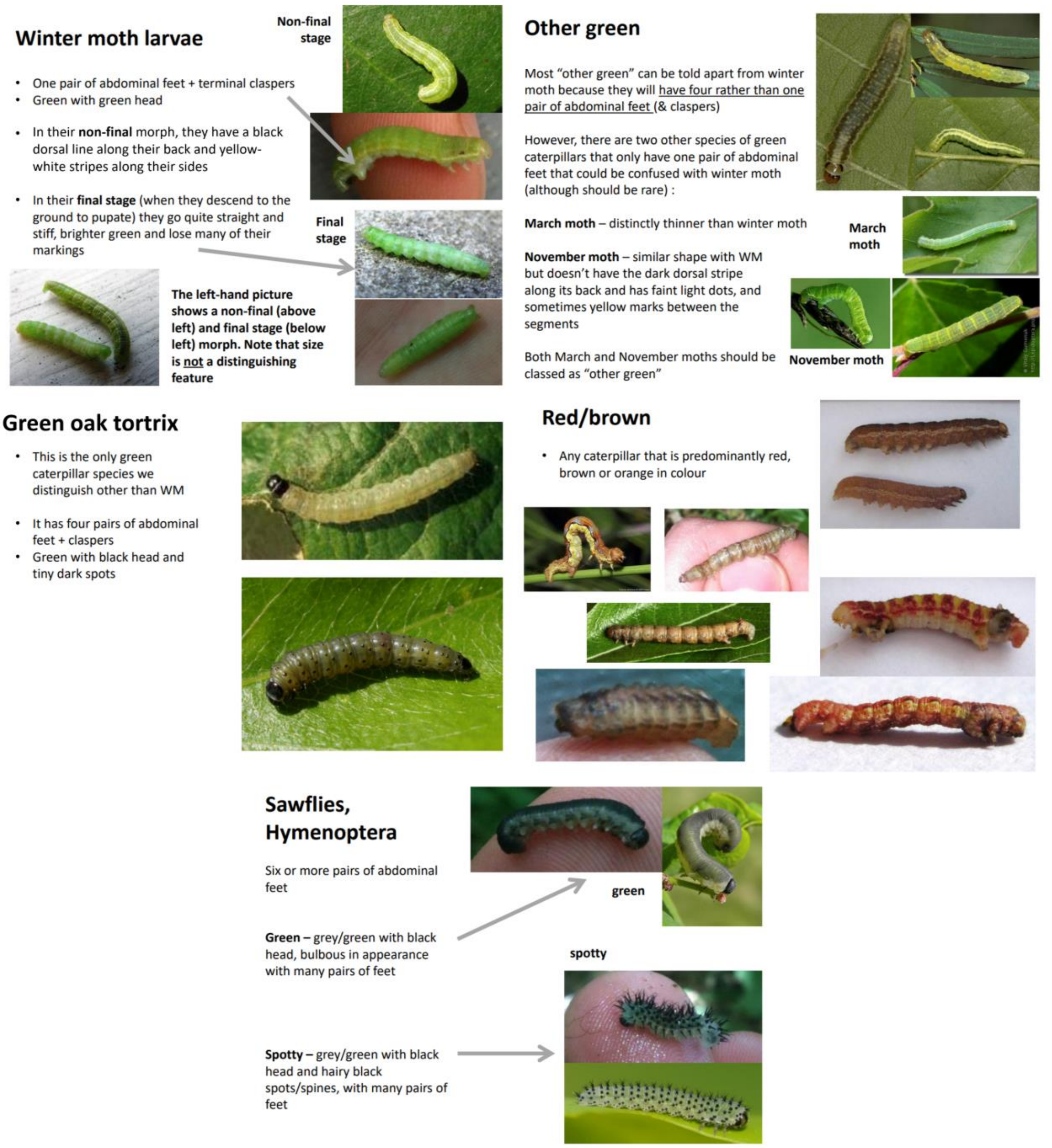

Supplementary Figure 2. Photographic reference keys used for identifying caterpillar groups. Images taken from Google Images.

Species	Metric	Mean Day (±sd)	Mean CI Width (±sd)	n	Rank
Ash	Budburst	123.76 (3.34)	5.11 (1.08)	23	6
	Half-Leaf	131.96 (4.05)	8.56 (1.54)	23	6
	Half-NDVI	126.21 (2.87)	7.64 (2.13)	22	5
	Frass Half-Fall	143.23 (2.23)	3.96 (0.74)	23	6
	TC Half-Fall	146.78 (3.37)	7.91 (3.13)	22	5
	FWM Half-Fall	149.10 (3.14)	14.18 (2.91)	12	6
	Frass Peak	143.14 (3.37)		23	4
	TC Peak	147.97 (5.08)		22	4
	FWM Peak	151.20 (5.03)		12	6
Beech	Budburst	110.79 (4.52)	10.67 (3.34)	5	4
	Half-Leaf	125.06 (1.24)	6.52 (1.98)	5	2
	Half-NDVI	121.37 (1.20)	4.85 (0.57)	5	4
	Frass Half-Fall	142.75 (1.25)	3.81 (0.31)	5	4
	TC Half-Fall	143.07 (4.24)	6.01 (2.26)	5	1
	FWM Half-Fall	143.97 (3.05)	12.12 (1.05)	4	1
	Frass Peak	143.95 (2.89)		5	6
	TC Peak	143.27 (3.91)		5	1
	FWM Peak	145.55 (3.30)		4	1
Birch	Budburst	100.33 (3.95)	6.84 (2.61)	17	2
	Half-Leaf	125.93 (5.07)	8.87 (1.75)	17	3
	Half-NDVI	115.25 (3.20)	8.64 (2.28)	16	2
	Frass Half-Fall	141.05 (1.83)	3.58 (0.69)	17	2
	TC Half-Fall	144.09 (4.11)	5.88 (0.10)	16	2
	FWM Half-Fall	146.31 (2.39)	8.97 (2.90)	14	3
	Frass Peak	140.41 (1.40)		17	1
	TC Peak	147.42 (5.34)		16	3
	FWM Peak	149.63 (4.60)		14	4
Hazel	Budburst	91.86 (5.36)	11.52 (2.63)	26 (CI n=6)*	1
	Half-Leaf	127.28 (3.72)	7.45 (1.34)	30	5
	Half-NDVI	NA	NA	NA	NA
	Frass Half-Fall	141.58 (1.93)	3.25 (0.63)	30	3
	TC Half-Fall	146.69 (3.22)	7.48 (2.23)	30	4
	FWM Half-Fall	148.33 (2.43)	11.15 (2.67)	24	4
	Frass Peak	141.59 (1.96)		30	3
	TC Peak	149.76 (5.62)		30	5
	FWM Peak	150.37 (4.17)		24	5
Oak	Budburst	111.88 (5.50)	11.07 (5.28)	77	5
	Half-Leaf	125.94 (4.38)	6.90 (1.42)	77	4
	Half-NDVI	121.27 (2.82)	5.88 (1.33)	76	3
	Frass Half-Fall	140.73 (2.04)	1.68 (0.35)	77	1
	TC Half-Fall	145.03 (2.21)	2.20 (0.39)	77	3
	FWM Half-Fall	145.42 (2.56)	5.84 (1.30)	76	2
	Frass Peak	141.24 (3.27)		77	2
	TC Peak	146.83 (3.89)		77	2
	FWM Peak	147.70 (4.98)		76	2
Sycamore	Budburst	102.86 (6.85)	8.20 (2.75)	18 (CI n=17)*	3
	Half-Leaf	123.56 (3.11)	6.69 (0.70)	18	1
	Half-NDVI	114.15 (4.16)	6.52 (1.45)	15	1
	Frass Half-Fall	142.83 (2.28)	2.51 (0.33)	18	5
	TC Half-Fall	147.61 (2.14)	3.91 (0.70)	17	6
	FWM Half-Fall	148.78 (2.95)	11.09 (2.39)	17	5
	Frass Peak	143.18 (2.51)		18	5
	TC Peak	150.55 (5.04)		17	6
	FWM Peak	149.30 (5.01)		17	3

Supplementary Table 2. Means and standard deviations of day values for nine phenological metrics for each tree species across 170 trees sampled in Wytham Woods. Rank denotes the order in which species reach their mean date for each metric, 1 being earliest, 6 being latest (5 for half-NDVI as hazel does not have NDVI data). Reduction in sample size for hazel budburst is due to four trees having curves that would cross y=0.5 earlier than the start of sampling, likewise for the lower sample size in hazel and sycamore budburst CIs as the lower bounds were outside of day range. Lower sample sizes in NDVI occur as we were unable to delineate a minority of tree crowns, whilst lower sample sizes in caterpillar metrics occur when water traps for certain trees never contained any caterpillars or specifically final-instar winter moths.

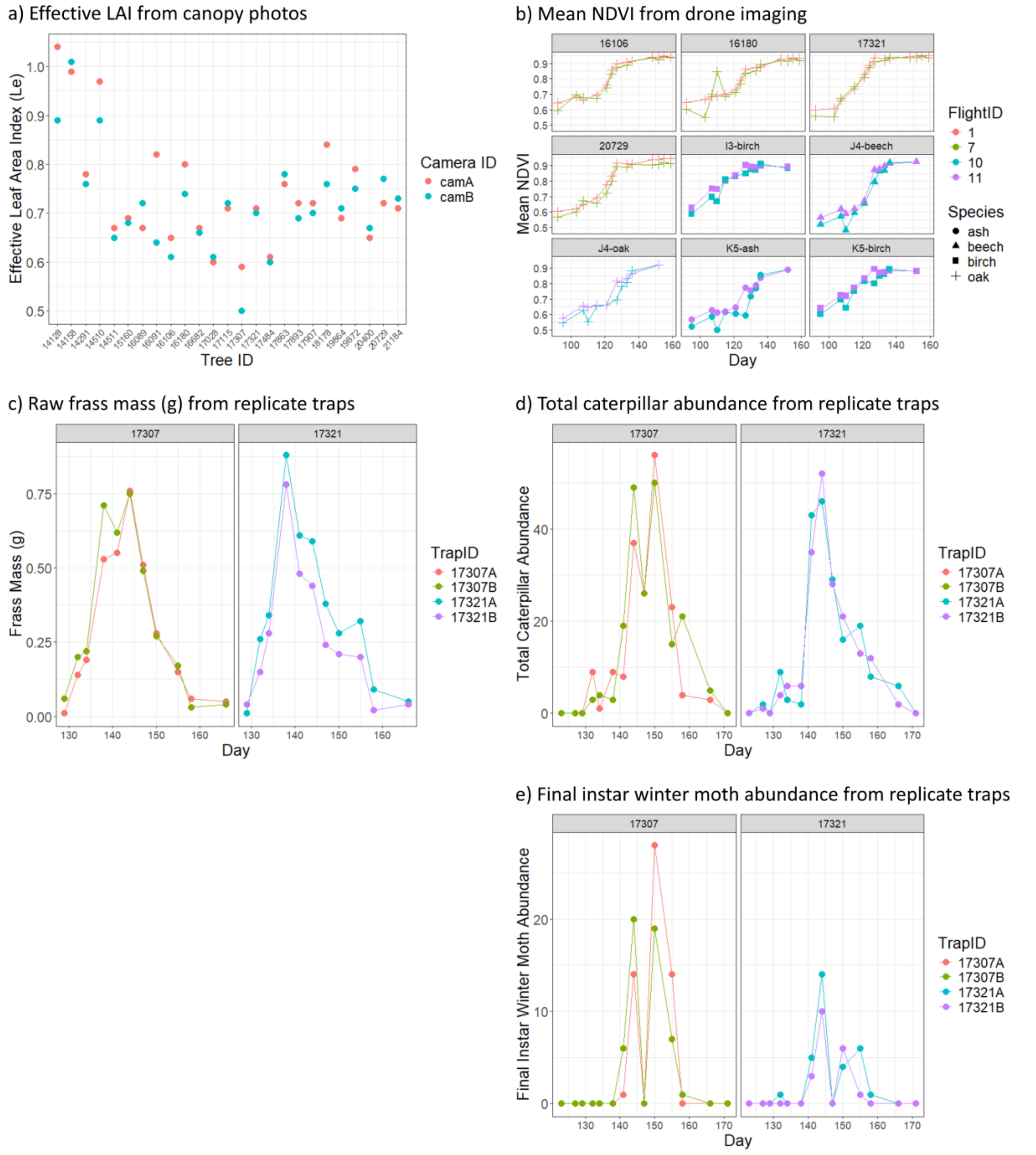

Supplementary Figure 3. Visualisations of repeated measurements made to assess repeatability of four out of the five field methods. **a)** The 25 ForestGEO oaks were imaged by two identical camera setups, one immediately after the other, on 28th March 2023. **b)** Nine trees were within overlapping flights so two sets of mean NDVI values existed for when the two flights occurred on the same day. **c)** Two oak trees had two frass traps beneath them across the whole season, and similarly the same two trees also had two replicate water traps to give two sets of **d)** total caterpillar and **e)** final instar winter moth counts.

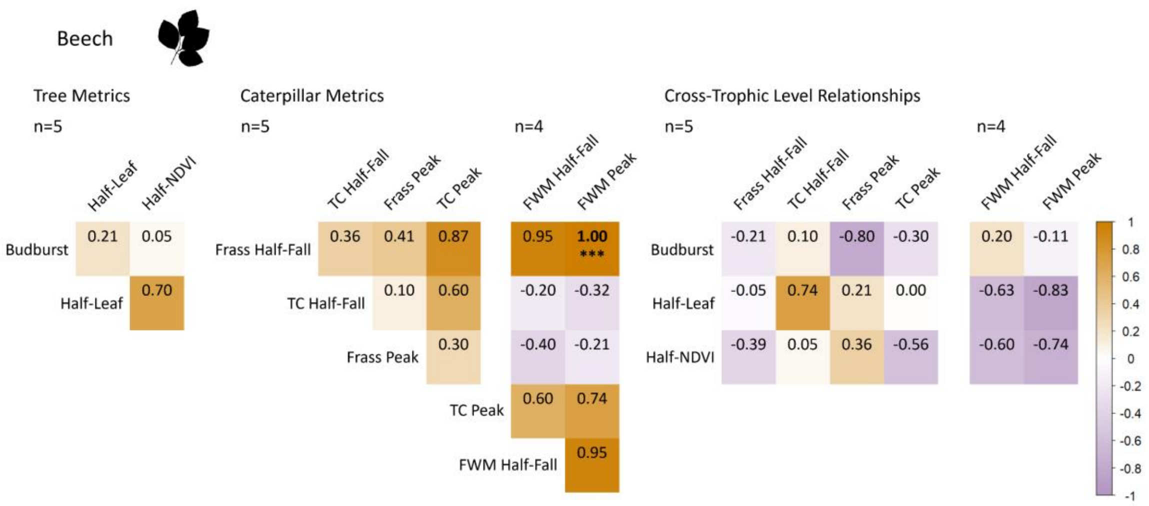

Supplementary Figure 4. Correlation matrix between the nine phenological metrics for beech trees. Values are Spearman correlation coefficients; those in bold are significant at the p<0.05 threshold and asterisks denote significance thresholds: * = p<0.05, ** = p<0.01, *** = p<0.001. Differing sample sizes between occur as one tree did not collect any final-instar winter moths.

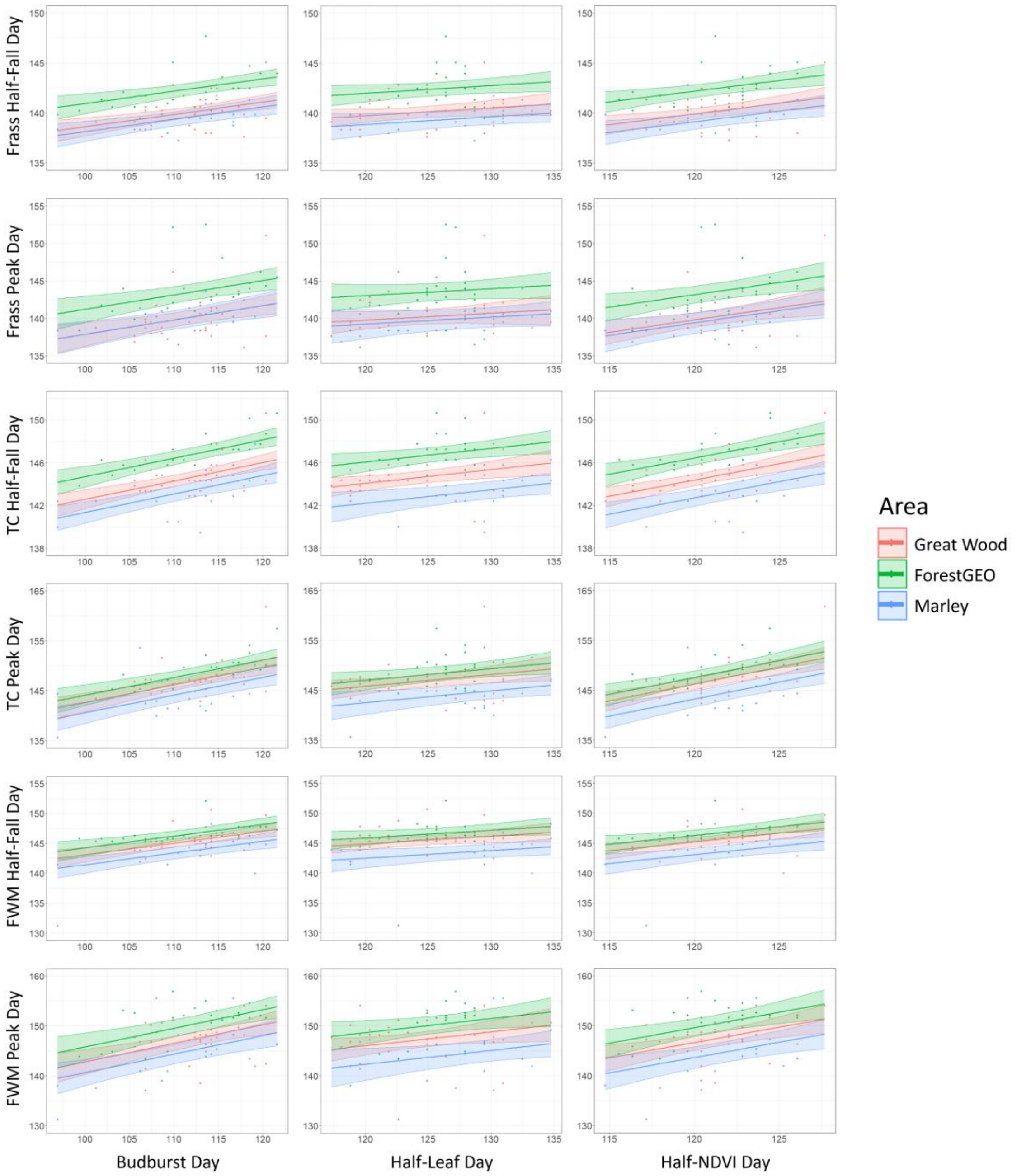

Supplementary Figure 5. Conditional effects plots of models quantifying relationships between three tree phenology metrics (columns) and six caterpillar phenology metrics (rows) for 77 oak trees in Wytham Woods, across sampling areas. Shaded areas are 95% credible intervals. Note that x-axis scales are consistent within tree metrics and y-axis scales are consistent within caterpillar metrics. Coefficients, R^2 and statistical significance of these relationships are presented in **Table 1**. TC = total caterpillars, FWM = final instar winter moths.

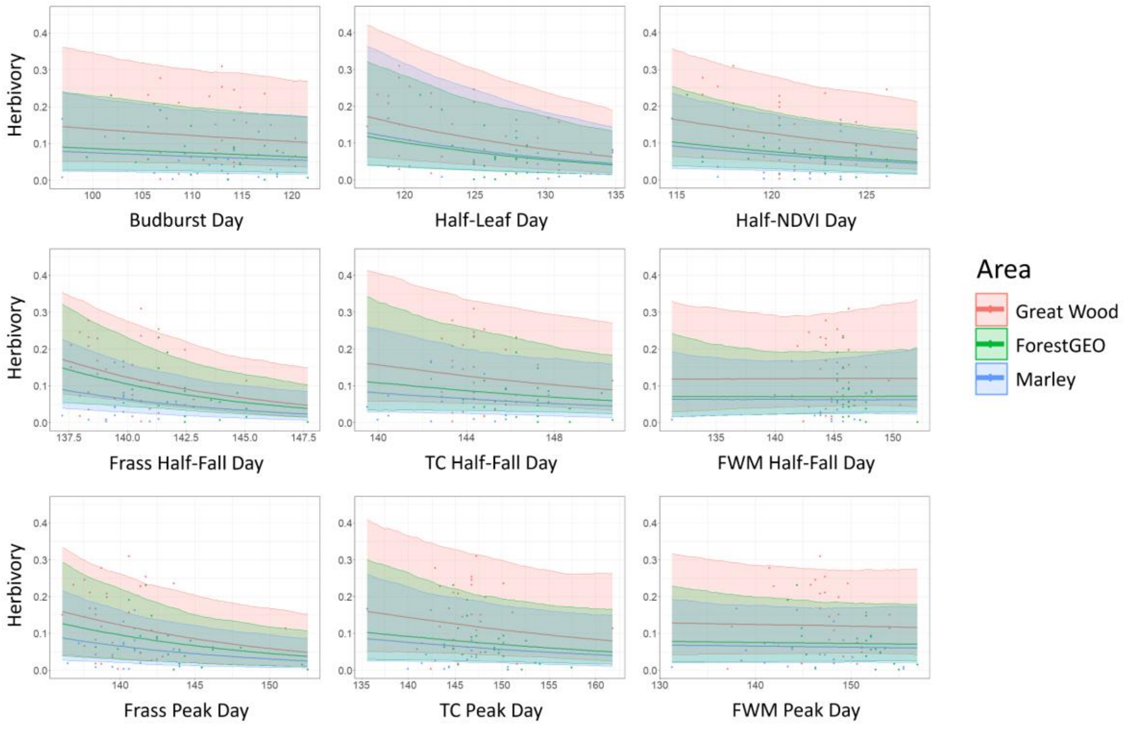

Supplementary Figure 6. Conditional effects plots of models quantifying relationships between herbivory and nine phenology metrics of 72 oak trees in Wytham Woods, across sampling areas. Shaded areas are 95% credible intervals. Note that the y-axis scale is consistent between plots but the x-axis scale changes between metrics. Coefficients, R^2 and statistical significance of these relationships are presented in **Table 2**. TC = total caterpillars, FWM = final instar winter moths.
